# ^1^H-NMR and LC-MS Based Metabolomics Analysis of Potato (*Solanum tuberosum* L.) Cultivars Irrigated with Fly Ash Treated Acid Mine Drainage

**DOI:** 10.3390/molecules27041187

**Published:** 2022-02-10

**Authors:** Maropeng V. Raletsena, Samukelisiwe Mdlalose, Olusola S. Bodede, Hailemariam A. Assress, Adugna A. Woldesemayat, David M. Modise

**Affiliations:** 1 Department of Agriculture and Animal Health, College of Agriculture and Environmental Sciences, Private Bag X6, Florida 1710, South Africa; samukelisiwe.mdlalose@mogalecity.gov.za (S.M.); ebodedos@unisa.ac.za (O.S.B.); 2 Arkansas Children’s Nutrition Center, 15 Children’s Way, Little Rock, AR 72202, USA; hmariam.abrha@aau.edu.et; 3 Department of Pediatrics, University of Arkansas for Medical Sciences, Little Rock, AR 72202, USA; 4 Genomics and Bioinformatics Research Unit, Department of Biotechnology, Addis Ababa Science and Technology University, Addis Ababa P.O. Box 16417, Ethiopia; adugna.abdi@aastu.edu.et; 5 Food Security and Safety (FSS), Faculty of Natural and Agricultural Sciences, North-West University, Private Bag X2046, Mahikeng 2735, South Africa; david.modise@nwu.ac.za

**Keywords:** acid mine drainage (AMD), fly ash (FA), metabolites, metabolomics, nuclear magnetic resonance (NMR), potato (*Solanum tuberosum* L.)

## Abstract

^1^H NMR and LC-MS, commonly used metabolomics analytical platforms, were used to annotate the metabolites found in potato (*Solanum tuberosum* L.) irrigated with four different treatments based on FA to AMD ratios, namely: control (0% AMD; tap water), 1:1 (50% AMD), 3:1 (75% AMD is 75% FA: AMD), and 100% AMD (untreated). The effects of stress on plants were illustrated by the primary metabolite shifts in the region from δ_H_ 0.0 to δ_H_ 4.0 and secondary metabolites peaks were prominent in the region ranging from δ_H_ 4.5 to δ_H_ 8.0. The 1:3 irrigation treatment enabled, in two potato cultivars, the production of significantly high concentrations of secondary metabolites due to the 75% FA: AMD content in the irrigation mixture, which induced stress. The findings suggested that 1:1 irrigation treatment induced production of lower amounts of secondary metabolites in all crops compared to crops irrigated with untreated acid mine drainage treatment and with other FA-treated AMD solutions.

## 1. Introduction

Acid mine drainage is a side effect of mining operations all over the world [1]. This mine effluent, which is both chemically toxic and radioactive (in the case of the West Rand), is generated from mines in several ways: as run-off from mine dumps entering surface water streams and groundwater, seepage from mine dumps into underground water, and as overflow from abandoned mines [2,3,4,5,6]. What makes the problem of AMD unique and particularly problematic, is the fact that it is extremely difficult to rectify and has the potential to persist for centuries, even after mine closure, as attested by [7]. Irrigated agricultural produce often experiences a variety of negative effects caused by changes in the supply and quantity of irrigation water, including reduced yields, deteriorated quality, and soil quality degradation [8]. Researchers [1,9,10,11] have recommended different methods of ameliorating acid mine drainage so that it can be utilized as irrigation water for crop production, and these include the use of lime, phosphorous rock, artificial wetlands, and the reverse osmosis process. The cost of the methods used in decontaminating AMD water varies, as some, such as fly ash (FA), are less expensive and can boost the irrigation water supply compared to liming, which is expensive [5]. Given that it is relatively cheaper, the use of FA to treat AMD may alleviate water shortages that the South African agriculture industry is experiencing. In fact, previous research has suggested that treating AMD with FA could be a feasible alternative to liming [12]. ESKOM, South Africa’s largest energy provider, produces most of this by-product (about 20 Mt of FA every year) [13]. While the rest of the world has made tremendous progress in adopting environmentally friendly technology to combat mining pollution and poverty alleviation, Africa, particularly Sub-Saharan Africa, has continued to lag [14,15]. South Africa is burdened by a water security crisis, even though the country’s economy is fueled by a thriving mining industry [16]. As a result of the spilling of highly acidic water into the country’s water system, these two phenomena have caused harm to communities, as well as ecosystems [15,16]. The contaminated water released from abandoned mines poses a concern to nearby residential populations, particularly those living along the Vaal and Limpopo rivers [17,18,19]. This concerning condition, along with the threat of climate change to food security, necessitates the use of less expensive, readily available and ecologically friendly solutions to address the country’s crisis. This research seeks to establish the suitability of AMD for the irrigation of potatoes and to establish the effects that this kind of water has on the biochemical composition and physiological aspects of the crop and on its rhizosphere. The main aim of this research is to determine if, despite the obvious detrimental effects of AMD, there could be measures to reduce its toxicity and render it useful for plant production through an innovative way of treating it for use in irrigation.

### 1.1. Shortfalls of Some Methods of Ameliorating Acid Mine Drainage Water

Although several acid mine water treatment techniques and methods exist, they all have certain disadvantages, such as the high cost incurred and challenges with huge precipitation leading to insolubility [20,21]. Phosphate rock for example has been used in some studies to control AMD. The phosphate usually costs much more than other calcium-based amendments and is needed in about the same amounts [22]. Unfortunately, on the other hand, the successful application of limestone is limited due to its low solubility and tendency to develop an external coating, or armor of ferric hydroxide (Fe(OH)_3_) when added to AMD [9,10]. Wetlands, on the other hand, are generally recommended to ameliorate heavy metals in contaminated water. There are three main processes of a wetland system namely (1) soil and substrate, (2) hydrology, and (3) vegetation. The complete process is dependent on each other, thus making the whole process of heavy metal removal mechanisms in wetlands very complicated and the vegetation gets negatively affected by heavy metals [23].

### 1.2. The Case for Use of AMD and Fly Ash for Crop Production: Importance of Utilizing Fly Ash (FA)

Wang et al. [24] suggested that fly ash can be utilized as an adsorbent for the removal of typical dyes from aqueous solutions. Many possible beneficial applications of fly ash have been evaluated to minimize waste, decrease the cost of disposal, and provide value-added products [25]. It was thus found to be advantageous as an ameliorant, as it could recover the physical, chemical, and biological properties of problem soils, and it also contains readily available macro-and micronutrients for uptake by plants. Studies by [25,26] confirmed that when fly ash is utilized in degraded soil, it enhances biomass production by plants. They further added that FA is a useful soil ameliorant and that its properties improve the fertility and productivity of soils. Rios et al. [27,28] are among researchers that have carried out extensive studies to indicate that fly ash can be used successfully in the purification of water and the removal of heavy metals from the polluted water. They also confirmed that it is a valuable source of essential plant nutrients (e.g., Ca, Mg, K, P, S, B, Fe, Cu, and Zn). Materials contained in fly ash have also been regarded as a potential resource for related agricultural activities as well as other industrial purposes. For example, in agriculture, it is valued as an applicable and non-toxic fertilizer or soil amendment that can be utilized to cleanse soil [29]. Many other researchers agree with this notion [4,5,30,31]. The properties are very important as there are concerns regarding future supplies of lime especially that reserves may be consumed by 2050 [25]. The addition of more sodium changes the composition of water from neutral to alkaline [32]. This principle is applicable in the present research with regards to the treatment of AMD, as well as the use of fly ash. Shi et al. [33] proved that heavy metals can form solutions in water, even though some may be toxic when in solution. According to [27], water dilution uses the principle that when water is added to certain elements, for example, sodium (Na), based on the concentration of Na ions, NaOH is formed. The above literature refers to various strategies that have been used previously to ameliorate undesirable effects of AMD water; however, none of the strategies involve the treatment of AMD in order to assess its use in crop production. This research proposes mixing AMD water with FA at different concentrations. This approach is scantly researched, if at all, and is not currently documented in the published literature. It is envisaged that the detrimental effects of AMD would be reduced through the use of the water dilution approach, and the product of this approach can be used to irrigate crops that would be acceptable for human consumption and would have minimal environmental concerns. Recent research work by [34] confirmed that AMD contains significant quantities of heavy metals, and fly ash was used to ameliorate the concentrations. Earlier, other researchers [35,36] found elevated concentrations of cadmium, cobalt, copper, lithium, mercury, strontium, and zinc that they believed lead to the formation of necrotic spots associated with a drastic degradation of Rubisco, especially Rubisco LSU, in rice leaves. Researchers, such as the authors of [37], have long carried work to investigate nutrient and metabolomic profiling (molecular) components in various plants. Currently, there are no published reports on the effects of heavy metals (treated or untreated with fly ash) on metabolites in potatoes.

Potatoes (*Solanum tuberosum* L.) are the world’s fourth most important food crop, following rice, wheat, and maize, and is the only major tuber crop [38]. In comparison to cereals, potatoes are a particularly effective food crop, producing more dry matter, protein, and minerals per unit area. Potatoes, which are a staple food in affluent countries, provide 130 kcal of energy per person per day, compared to 41 kcal in underdeveloped countries, where they are still classified as a vegetable. Potatoes, in addition to being a strong source of starch, include many small molecules and secondary metabolites that are involved in a variety of biological processes [39]. Many of the compounds found in potatoes are important because of their health benefits, making them an excellent addition to the human diet [40,41]. Nutritional deficits are not generally known in areas where potatoes are the main source in diet [42]. Increased nutrition availability for a substantial portion of the world’s population is a global health goal. Increasing the nutritious value of commonly consumed crops would be a practical way to attain this goal. Potatoes are farmed and consumed in great amounts all over the world [38]. Other researchers [41] described metabolomics as a molecular phenotype. This is further described as the large-scale study of small molecules, commonly known as metabolites, within cells, biofluids, tissues, or organisms.

The metabolome is regarded collectively as small molecules and their interactions within a biological system. Studies [43] have regarded metabolomics as a powerful approach because metabolites and their concentrations, unlike other “omics” measures, directly reflect the underlying biochemical activity and state of cells. Other work [44] confirmed that metabolites, like genes or gene clusters, play a role in plant growth, development, and response to environments. Metabolomics, together with other omics, allows us to solve key problems of agronomic performance. A study has never been done where fly ash-treated acid mine drainage water was used the potato crops; nonetheless, the molecular mechanism signaling methods were addressed in the current study. Chromium toxicity can trigger three types of metabolic disturbances: (1) alterations in pigment production, (2) increased metabolite production (ascorbic acid, glutathione), and (3) production of new metabolites that make a plant tolerant to chromium toxicity. The structure of the plant root tissues is competent in filtering or ejecting several heavy metals; if the soil has a higher concentration of heavy metals, then it will be toxic to plants. Nickel concentration has also been studied in some potato cultivars, and it was proven that nickel suppresses growth and decreased iron levels. Thus, nickel can substitute iron in the plant. Nevertheless, a combination of cadmium and zinc was also proven to decrease the levels of ascorbic acid, total phenols, carotenoids, and chlorophyll content [45]. Previous research was done on heavy metals and salt stress; it was proven that abiotic stress causes decreased levels of several metabolites, which may exist before and after folding in the plant cell [46,47]. The literature shows that heavy metals from acid mine drainage water have an impact on the metabolome of any given cell. Little or no work has been done on the effects of acid mine drainage water treated with fly ash; hence, this research must be conducted. In terms of detection and sensitivity, LC-MS and NMR are two different analytical techniques [47]. When compared to NMR, LC-MS is a faster and more sensitive technology. However, metabolite separation is dependent on the chromatographic column used, detection is limited by the analytes’ ionization ability, and molecular elucidation has some inherent limits, such as isomer resolution [48]. Given that analytes are soluble, NMR is unaffected by matrix characteristics. When compared to MS, NMR is a highly selective technology for discriminating molecular structures, although it has a lesser sensitivity [49]. Therefore, the present study is conducted to understand the metabolome changes across a potato crop (tubers) irrigated with acid mine drainage water to comprehend the molecular mechanisms and signaling pathways.

## 2. Materials and Methods

### 2.1. Potato (Solanum tuberosum *L.*) Tuber Samples and Experimental Design

The study was conducted over two growing seasons (April to July 2018 (Season 1) and October to December 2018 (Season 2). The plants were grown in a greenhouse-controlled average temperature of 15–30 °C at the Florida Science campus of the University of South Africa (26°10′30″ S, 27°55′22.8″ E). Soil analysis was performed at the Agricultural Research Council, Institute for Soil, Climate and Water (ARC-ISWC) in Pretoria (25°44′19.4″ S, 28°12′26.4″ E). Sterilized 3:1:1 growth media (topsoil, river sand, and vermiculite) was used. Certified seeds of two cultivars, *Lady Rosetta* (determinate) and *Fianna* (indeterminate), were obtained from First Potato Dynamics in Western Cape Province and were stored at the National Potato (Nationale Aartappelkantoor (NAK)). The storage facilities were in Bethal in Mpumalanga Province All seed tubers were first stored at a temperature of 3 °C and later increased to 15 °C in 14-day intervals. The pot experiment was a completely randomized design with five (5) replicates per treatment and the factorial design was indicated. The pots were spaced 35 cm apart. Each block comprised 12 plants in pots, resulting in 48 plants per block. A total of 144 plants were used for the experiment. The treatments constituted a control, 50% FA: AMD, 75% FA: AMD ratio, and, 100% AMD (untreated AMD). Plants were well irrigated before starting with the treatments. Irrigation treatments were executed two weeks after seedling establishment. Harvesting was done upon maturity of the plants, at 90 days after planting [40].

### 2.2. Sample Preparation for NMR Analysis

The representative potato tuber samples (72) were subjected to metabolomic analyses. Untargeted metabolites were extracted from potato tuber tissues using an extraction system of methanol/water (1/1), as described previously [50,51], with modification according to [52]. The tissue sample (approximately 300 mg) was ground in a liquid N_2_-cooled mortar and pestle. The tissue powder was thoroughly homogenized in 3.33 mL g^−1^ methanol/water (1/1) using a high throughput homogenizer, Precellys 24 (Bertin technologies SAS, avenue Ampère, France). After homogenization, the sample was transferred into an Eppendorf tube and centrifuged for 10 min (3000× *g*, 4 °C). The supernatant was removed and then lyophilized. It was subsequently re-dissolved in 600-µL phosphate buffer (0.1 M Na_2_HPO_4_ and NaH_2_PO_4_, containing 0.5 mM TSP, pH 7.0) in D_2_O. The mixture was then vortexed and centrifuged at 3000× *g* for 5 min at 4 °C. The supernatant substance (650 µL) was pipetted into a 5 mm NMR tube for ^1^H NMR spectroscopic analysis.

### 2.3. LC-MS Experiments

A total of 0.5 g fresh weight of plant material was weighed and added to 1.5 mL of MeOH (about 75% MeOH and 25% water). Another 5 mg dry weight of plant material was weighed and added to 1.5 mL of MeOH (about 75% MeOH and 25% water). When the signal/noise in triple quad MS or LC-Impact II-MS was not ideal, a total of 10 mg dry weight material was added. The sample mixture was sonicated for 5 min and centrifuged for 15 min (10,000 rpm table centrifuge). The supernatant was filtered through 0.2-micron syringe filters (Labotec, Midrand, South Africa) with a 1 mL plastic discardable pipette. Filtration was considered to avoid large particles, which can ruin the injector and column of the UPLC. A total of 0.5 mL of filtrated sample was dried using a speed vac. The sample was re-suspended (after dryness on speed vac) with 0.5 mL 100% ultrapure water and sonicated for 5 min, centrifuged, and the supernatant transferred into an HPLC vial (for LC-QQQ-MS analysis, triple quad MS). The remaining filtrate was transferred into another HPLC vial (for LC-Impact II-MS analysis).

The separation of analytes was carried out using a Dionex Ultimate 3000 UHPLC system (Dionex Softron GmbH, Germany) equipped with a reversed-phase C18 analytical column of 100 mm × 2.1 mm and 1.7 µm particle size (Acquity UPLC^®^ BEH, Waters, Ireland). The column temperature was maintained at 35 °C. The injected sample volume was 5 µL. Mobile phases A and B were water and methanol with 0.1% formic acid, respectively. The optimized chromatographic method was programmed as follows: the initial mobile phase composition (2% B) was held constant for 1 min, followed by a linear gradient from 2% B to 100% B for 9 min, kept at 100% B for 2 min, and then dropped back to 2% B for 12.1 mins and kept constant at 2% B for 2 min. The flowrate used was 0.3 mL/min and the total run time was 14 min.

This UHPLC system was connected to an ultrahigh-resolution quadrupole time-of-flight mass spectrometer (Impact II Bruker, Bruker Daltonics GmbH, Bremen, Germany) equipped with electrospray ionization, operating in positive ion mode. LC/MS accurate mass spectra were recorded across the range of 50–1600 *m*/*z*. The recorded data were processed using Bruker Compass Data Analysis 4.3 software (https://www.bruker.com/en/products-and-solutions/mass-spectrometry/ms-software.html accessed on 19 December 2021). Accurate mass measurements of each peak from the extracted ion chromatograms were obtained using a sodium formate calibrant solution delivered by a KdScientific external pump. The instrument was operated in full-scan mode, except in cases where automated MS-MS was necessary to discriminate isobars/isomers, as well as for identification of selected compounds and degradation products, as explained in the results.

### 2.4. Data Pre-Processing and Multivariate Analysis

All NMR spectra were phase-corrected, and baseline corrected using Mestrenova version 10.1 (Mestrelab Research). The region containing the water and methanol (4.75–5.1 ppm and 3.28–3.33 ppm) were excluded before statistical analyses. The remaining spectral regions were divided into 0.04 ppm bins, saved as ASCII files, and then imported into Excel for statistical analyses using SIMCA. PCA is an exploratory unsupervised pattern recognition method of analysis, which is blind to the status of each sample and serves to reduce the dimensionality of the data and summarize the similarities and differences between the control and treatment groups [53]. The algorithm of this pattern recognition method calculates the highest amount of correlated variation along PC1, with subsequent PCs containing correspondingly smaller amounts of variance. For each built model, the loading vector for the PC was examined to identify the metabolites that contributed to the clusters. The Excel file was then imported to SIMCA 15 for statistical analyses. The principal component analysis (PCA) was initially performed to identify, based on the presence of any intrinsic variation between the samples, if there were any extreme outliers. After unsupervised PCA, supervised orthogonal partial least square-discriminant analysis (OPLS-DA) was performed and the metabolites contributing to variations were annotated from the loading plots derived from the OPLS-DA scores.

## 3. Results

### ^1^H-NMR Analysis

**Figure 1:**
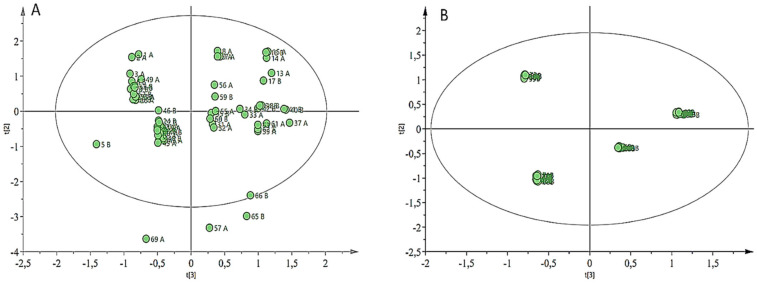
An example of a PCA-X model of the *Solanum tuberosum* L. tuber samples irrigated with treated and untreated acid mine drainage. This PCA-X model provides an overview of the potato crop metabolite profile measured in season one, April to July 2018 (**A**) and season two, October to December 2018 (**B**).

Figure 1 depicts PCA analysis of ^1^H-NMR spectra of two potato crops irrigated with acid mine drainage. Each point in the PCA scatter plot is a demonstration of an individual sample. The samples in the left pane are cultivar 1 (Fianna) and the samples in the right pane represent cultivar 2 (Lady Rosetta). In addition, the model showed a good fit (R^2^X (cum) = 0.67), (RY (cum) = 0.95) and predictive ability (Q2 (cum) = 0.58). Some of the samples showed as negative along PC1 and PC2, except for one outlier for cultivar 1 (Fianna) and two outliers of cultivar 2 (Lady Rosetta).

**Figure 2:**
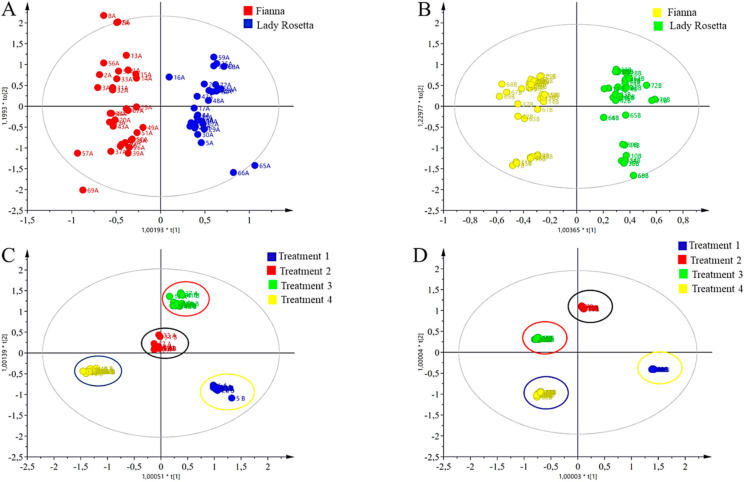
OPLS-DA score plot of the potato tuber samples irrigated with treated and untreated acid mine drainage for season one; April to July 2018 (**A**,**C**). The cluster in red represents cultivar 1 (Fianna) while those in blue represent cultivar 2 (Lady Rosetta). OPLS-DA score plot of the potato tuber samples irrigated with treated and untreated acid mine drainage for season two, October to December 2018 (**B**,**D**). The cluster in yellow represents cultivar 1 (Fianna) while those in green represent cultivar 2 (Lady Rosetta).

To improve the clustering and to identify the metabolites responsible for the differences between the AMD-treated samples, the OPLS-DA model was constructed (Figure 2A,B) and revealed RY^2^ and Q^2^ values of 0.84, 0.89, and 0.84, 0.63, respectively, for both seasons. The samples from all AMD-treated plants were separated in the OPLS-DA score plot, which means that the non-correlated variations in X metabolites to Y metabolites were removed, resulting in maximum separation.

Generally, all AMD treatments presented metabolites as being unique to each. The sugar region in samples irrigated with the control was unique, in that the differences were observed even on the spectra itself before statistical analysis with SIMCA, with four components with R^2^X and Q^2^ values of 0.83 and 0.99, respectively, being observed. There was a distinct difference when comparing all samples irrigated with different levels of AMD. The blue cluster encircled in yellow represents tuber samples of the control treatment. The red cluster encircled in black represents tuber samples treated with 1:1 FA:AMD treatment. The green cluster encircled in red represents the samples that were irrigated with 3:1 FA:AMD treatment. The yellow cluster encircled in blue represents samples that were irrigated with untreated acid mine drainage (0:1, FA:AMD treatment). The treatment proved that there was a significant difference in all samples, hence they grouped and no outliers were observed from the OPLS-DA (Figure 2C,D). For the OPLS-DA model corresponding to *y*-axis, intercepts of R^2^ X = (0.0; 0.84) and R^2^ Y = (0.0; 89) were found.

**Figure 3:**
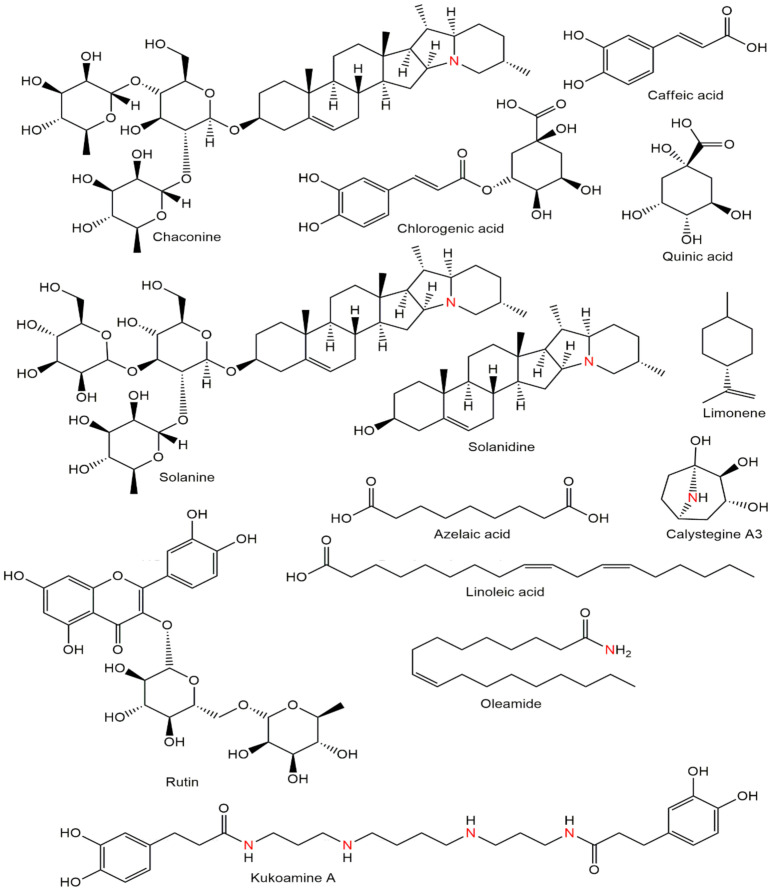
Metabolites were annotated by NMR from *Solanum tuberosum* L. (ChemSpider, https://www.chemspider.com/StructureSearch.aspx accessed on 19 December 2021).

Clustering into two groups (Fianna and Lady Rosetta) was observed, according to the cultivars, which indicated that there were some metabolites that were unique between the samples. Fianna and Lady Rosetta separated quite well and the greatest difference was observed since they were both further apart from the others. For season one (Figure 2A), cultivar one is in red and the second cultivar is in blue, respectively; the same results were observed in season two, wherein both cultivars separated quite well and were distinguished by yellow and green color coding, respectively. The sugar region in the peaks in the positive bars of the contribution plots of the season is more abundant than the aliphatic and aromatic regions, indicating the importance of sugars in the samples from season one than in season two. Using Chenomx, the metabolites were further annotated (Figure 3).

**Figure 4:**
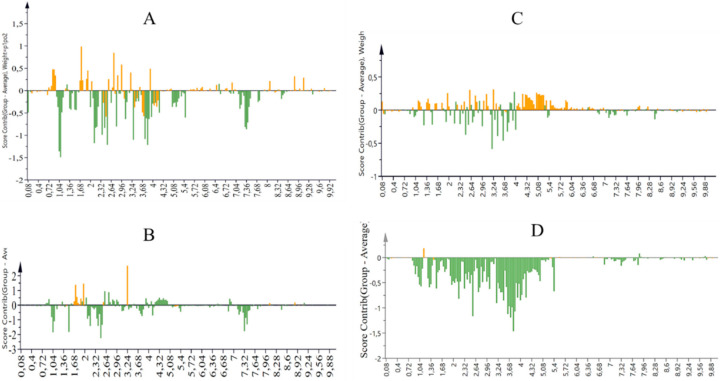
Contribution plots were generated by comparing two potato cultivars (Fianna and Lady Rosetta) irrigated with acid mine drainage for season one, April to July 2018 (**A**,**C**) and season two, October to December 2018 (**B**,**D**).

Contribution plots were then generated by comparing samples irrigated with the control to those irrigated with treatment two, three, and four. The buckets (positive bars) represent the specific regions of the *Solanum tuberosum* L. NMR spectra responsible for the differences. The negative bars represent the areas important in the other three FA:AMD ratios while the positive bars represent that the ones important in treatment 1 appeared to have more aromatics as the metabolites of importance in the control (Figure 4). The other two treatments (50% AMD, and 75% AMD) presented aliphatics as the important metabolites, as opposed to aromatics in treatment four (100% AMD). All metabolites annotated in this study were previously identified [54]. There is a great difference in the levels of metabolites between the treatments; therefore, the compounds of this plant irrigated with different levels of AMD are observed to differ.

**Figure 5:**
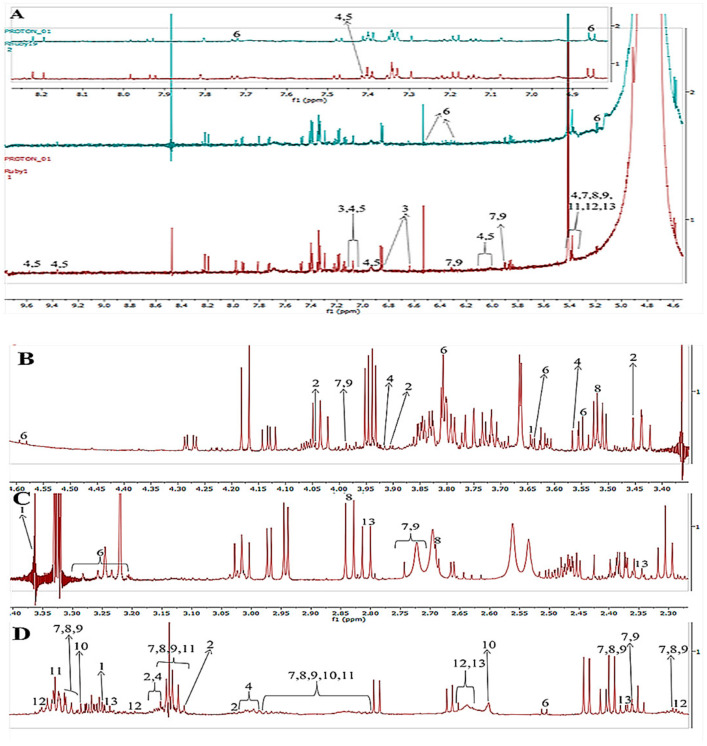
Representative ^1^H NMR spectra (expanded) for *Solanum tuberosum* L. irrigated with FA:AMD treatments. (**A**) δ_H_ 4.6–9.6, (**B**) δ_H_ 3.4–4.6, (**C**) δ_H_ 2.3–3.4, (**D**) δ_H_ 0.9–2.2.

Two clusters (Fianna and Lady Rosetta) were observed in the OPLS-DA, indicating the uniqueness of some metabolites between the samples. Fianna and Lady Rosetta separated quite well. For season one (Figure 2A), the red and blue represent Fianna and Lady Rosetta, respectively, while for season two (Figure 2B) had Fianna and Lady Rosetta clustered into yellow and green, respectively. Nevertheless, the contribution plots were constructed for both potato cultivars and it was evident that the sugars contributed to the differences between Fianna and Lady Rosetta (Figure 4A,B). The sugar peaks of the contribution plots of season one are more abundant than the aliphatic and the aromatic regions, indicating the importance of sugars in the season one samples, compared to those in season two. Using Chenomx, the metabolites were further annotated (Figure 5). The contribution plot was generated by comparing the two potato cultivars (Fianna and Lady Rosetta) irrigated with acid mine drainage for season one (April to July 2018). Figure 2B represents the contribution plot of OPLS-DA for cultivars of *Solanum tuberosum* L. tuber extracts (Q2 = 0.63). For the OPLS-DA model corresponding to *y*-axis, intercepts were R2 X = (0.0; 0.84) and R2 Y = (0.0; 89). The contribution plot was generated by comparing two potato cultivars (Fianna and Lady Rosetta) irrigated with acid mine drainage for season two (October to December 2018).

The assignments of ^1^H-NMR chemical shifts for all the additives encountered are presented in Table S1. More than 25 compounds were determined, of which 13 were annotated; the metabolites ranged from 0.90 to 10 ppm. Compounds 2.28 to 4.00 accounted for the highest abundance amongst the treatments (Figure 5B,C). Due to the time constraints and the scope of this study, it was not possible to annotate all metabolites. The presence of a metabolite at *m*/*z* 180; ^1^H NMR (CD_3_OD, 600 MHz) δ_H_: 7.55 (m), 7.07 (m), 6.95 (m), 6.81 (m), 6.24 (m) is annotated as caffeic acid. Caffeic acid is known as phenolic acid, which is the derivative of cinnamic and benzoic acids. These chemicals can be found in a wide range of foods. They have an impact on flavor, stability, nutritional value, color, and other food attributes, as is well known [55,56]. Because of their ability to scavenge free radicals, inhibit lipoxygenase, and chelate metals, phenolic acids have been found to play a variety of biological activities. Furthermore, it has been eluded that the consumption of phenolic acids through vegetables and fruits has a beneficial effect on health [57]. There are two types of phenolic acids: hydroxybenzoic acids and hydroxycinnamic acids. P-hydroxybenzoic, syringic, protocatechuic, gallic, and vanillic acids are all hydroxybenzoic acids. Sinapic, coumaric, ferulic, and caffeic acids are examples of hydroxycinnamic acids with a C6-C3 skeleton [54].

Calystegines are a new class of alkaloids with a nortropanic skeleton in common. Six compounds have been isolated so far [4]. These compounds were annotated by ^1^H NMR (CD_3_OD, 600 MHz), as δ_H_ 3.63 (m), 3.38 (m), and 2.06 (m). According to Figure 5B–D the compound calystegines was only annotated for three chemical shifts and the peaks are very small in all samples irrigated with FA:AMD ratios. It has been confirmed by previous studies [45,46,47,58] that calystegines compounds have only been detected in the Brassicaceae, Convolvulaceae, Erythroxylaceae, Moraceae, and Solanaceae families of plants thus far. It was further illustrated that sweet peppers, aubergines, and potatoes, for example, are members of plant families that are commonly consumed by humans [59].

Quinic acid was identified with the chemical formula C_7_H_12_O_6_; ^1^H NMR (CD_3_OD, 600 MHz) δ_H_ 4.04 (m), 3.91 (d), 3.45 (s), 1.91–1.96 (m), 1.88 (m), 1.76 (s). Chlorogenic acid (C_16_H_18_O_9_) has been annotated as well, ^1^H NMR (CD_3_OD, 600 MHz) δ_H_ 7.42 (m), 9.61 (m), 9.22 (s), 7.02 (m), 7.12 (m), 6.01 (m), 5.41 (d), 3.93 (s), 3.57 (s), 1.92–1.96 (m) and 1.71–1.76 (m). Chlorogenic acid (5-O-caffeoylquinic acid, 5CQA), a natural polyphenol that may be extracted from a variety of fruits and vegetables, is an ester produced between caffeic and quinic acids (Figure 5). This molecule is an essential secondary metabolite in plants that has a variety of roles [60]. Elevated concentrations of 5CQA in plants have been shown to improve UV radiation protection and increase microbial resistance [61]. Furthermore, 5CQA is a pest resistance component in ornamental plants [38,62,63]. 5CQA, like other dietary polyphenols, has antihypertensive, anticancer, antidiabetic, hypolipidemic, anti-inflammatory, and antioxidative characteristics, as well as antioxidative properties [61]. It has been noticed that acid mine drainage treatments did not affect the number of such compounds.

Compound 6 (Rutin) with the structural formula C_27_H_30_O_6_ was annotated by ^1^H NMR (CD_3_OD, 600 MHz), δ_H_ 7.73 (d), 6.87 (d), (d), 4.60 (d), 3.81 (m), 3.65 (d), 3.55 (s), 3.25 (m) and 1.15 (d). Flavonoids are by-products of plant secondary metabolism [64]. The antioxidant impact of these compounds has piqued the interest of the food and pharmaceutical industries [62,65]. It has been suggested by [65] that oxidative stress occurs when the normal redox state of cells is disrupted, resulting in the formation of free radicals, which can be harmful. Free radicals can interact with flavonoids to produce less-reactive compounds [66]. As a result, these chemicals can protect cell membranes and biomolecules (such as DNA, proteins, and lipids) against free radical damage [67,68].

Compounds 7 and 9 were identified by ^1^H NMR (CD_3_OD, 600 MHz), δ_H_ 6.32 (s), 5.89 (d), 5.37 (d), 3.98(d), 2.72–2.81 (m), 2.10–2.36 (m), 1.89–1.93 (m), 1.66–1.80 (m), 1.05(d), 0.97 (m) and 0.881–0.86 (m). It has been suggested that saponins α-chaconine and α-solanine are proven to be important contributors to overall off-taste in potato fiber isolates, despite previously being reported as off-taste chemicals in potato tubers [63].

Compound 8 (Solanidine) was annotated in different chemical shifts. The ^1^H NMR (CD_3_OD, 600 MHz) δ_H_ 5.35(d), 2.81–2.91 (m), 2.57–2.68 (m), 2.15–2.36 (m), 1.93–2.04 (m), 1.80–1.90 (m), 1.66–1.80 (m), 1.05–1.21 (m), 1.02 (s), and 0.92 (d). There is a lot of overlap in the resonances for chemical shifts less than 2 ppm (Figure 5D). This is to be expected, as these chemical shifts correlate to the protons of alkyl chains, methyl, and tertiary butyl groups, all of which are present in both additives and polyolefin, oligomers extracted with the additives. At chemical shifts above 2 ppm, however, where protons associate with most functional groups appear, there is less overlapping of the resonance signals. Figure 5A,B shows that the signals at 6–5 and 8 ppm indicate the presence of aromatic protons, which could be phenolic antioxidants, and the signal at 5.35 ppm could be a long chain alkene, such as an unsaturated fatty derivative. Many studies [69,70,71,72] have reported the health-promoting properties of a high dietary fiber intake in recent years, such as a significant reduction in cardiovascular-related mortality and incidence of coronary heart disease, type 2 diabetes, and colon cancer 1–4 by stimulating insulin sensitivity, cholesterol reduction, and blood lipids, among other things. In potato dietary fiber isolates, 1D/2D NMR allowed the identification of key off-taste compounds [70]. Saponins α-chaconine and α-solanine, which were previously identified as off-taste chemicals in potato tubers, were found to be important contributors to overall off-taste in potato fiber isolates [71,72].

NMR data for azelaic acid: ^1^H NMR (CD_3_OD, 600 MHz) δ_H_: 2.09 (m), 1.50–1.70 (m), and 1.25 (s) were annotated as compound 10. According to the literature [33,34], it was confirmed that *Solanum tuberosum* L. contains secondary metabolites for growth regulators. Authors further eluded that potato sprouts contained substances that inhibit the growth of both fungi and higher plants [73]. It is quite clear that a growing potato sprout must comprise growth promoters, such as auxins. Potato sprouts include substances that impact the development of cucumber roots and fungus, according to scientific evidence [74]. According to this study, the FA:AMD treatments did not negatively affect the growth regulator (azelaic acid).

NMR data for limonene: ^1^H NMR (CD_3_OD, 600 MHz) δ_H_: 5.4 (m), 2.16 (m), 1.86–1.96 (m) and 1.50–1.70 (m). The molecule has a methyl group on the cyclohexene ring at 1.6 ppm and a terminal methyl group at roughly 1.7 ppm, and is equivalent to limonene. The peaks at 1.5 ppm, 1.9 ppm, and 2.1 ppm are caused by protons located on the cyclohexene ring. The peak at 4.7 ppm is because of the protons located at the terminal double bond while the peak at 5.4 ppm is related to the proton at the double bond on the ring. The above chemical shifts are supported by [75,76,77].

NMR data for oleamide: ^1^H NMR (CD3OD, 600 MHz) δH: 5.4 (m), 2.17(d), 2.01 (m), 1.30–1.34 (m), 0.89 (m). Researchers have confirmed that potatoes have sleep-inducing lipids [78]. A significant and sophisticated body of study has been focused on the discovery of endogenous sleep-inducing chemicals [79]. Several compounds have been reported to play a role in sleep induction, including sleep-inducing peptide and prostaglandin D2, nevertheless, the molecular processes of this physiological process remain mostly unexplored [78].

**Figure 6:**
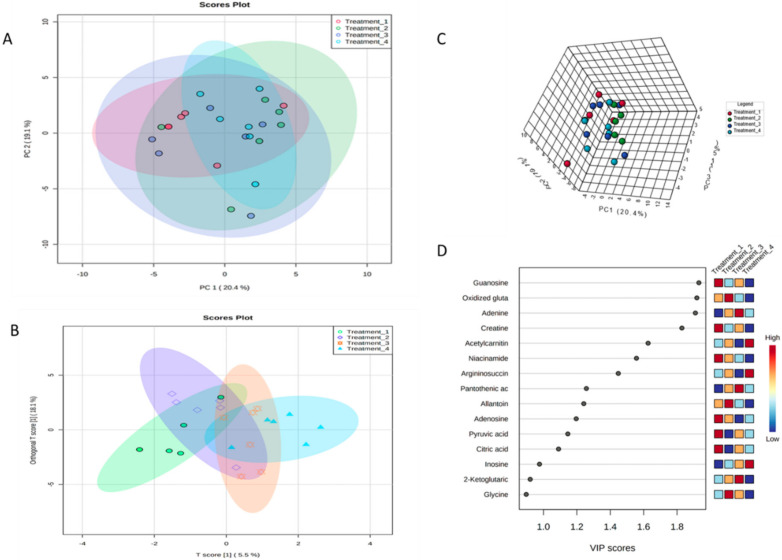
Score plot between selected PCs (**A**); 2D PLS-DA score plot between the selected PCs (**B**) 3D PLS-DA score plot between the selected PCs (**C**); important VIP scores identified by PLS-DA (**D**).

Even though different metabolite patterns were noted by visual inspection of LC-MS chromatograms in the samples irrigated with different FA:AMD ratios, we then analyzed the results more holistically using PCA to discover the relative variability in the samples irrigated with different ratios of AMD. All seventy-two samples were analyzed and no clear separation was achieved for both 2D and 3D PCA plots (Figure 6C). The samples did not separate, indicating that they were related in composition.

After unsupervised cluster analysis (Figure 6A) and supervised PLS-DA analysis (Figure 6B), among the 33 metabolites for *Solanum tuberosum* L. samples, 15 metabolites (guanosine, oxidized gluta, adenine, creatine, acetylcarnitine, niacinamide, argininosuccinic acid, pantothenic acid, allantoin, adenosine, pyruvic acid, citric acid, inosine, 2-ketoglutaric acid, and glycine) had VIP values above 1.0, which indicated a strong difference between the control and acid mine drainage treatments (50% AMD, 75% AMD and untreated AMD). Six metabolites (guanosine, creatine, niacinamide, adenosine, pyruvic acid, and citric acid) were significantly increased in the samples irrigated with the control, three metabolites (oxidized gluta, allantoin, and glycine) for samples irrigated with treatment two, three metabolites (adenine, pantothenic acid, and 2-ketoglutaric acid) for samples irrigated with treatment three, and three metabolites (acetylcarnitine, argininosuccinic acid, and inosine) were significantly increased in samples irrigated with treatment four. Some of the metabolites were present in high concentrations in the samples irrigated with the control, 1:1 (50% AMD), untreated AMD, and 1:3 (75% AMD). Figure 6D shows the VIP score for primary metabolites irrigated with FA:AMD treatments. According to Figure 2A, it is evident that some of the metabolites (acetylcarnitine, argininosuccinic, and inosine) are upregulated when potatoes are irrigated with untreated acid mine drainage when compared to the control. It has been reported by [80] that, in the roots of soybean (cultivar: *Enrei*) plants under Cr stress, the levels of adenosine, adenine, guanine, lysine, leucine, glycine, arginine, and arginine were dramatically enhanced, whereas glutamic acid and methionine were significantly decreased.

The 2D score plots (Figure 5B) did not show a clear separation, although the samples irrigated with FA:AMD treatments showed some clustering. The 3D plot (Figure 5C) showed that four distinct clusters were formed, distributed over three opposing regions on the PLS-DA plots corresponding to different AMD irrigations. Samples irrigated with untreated AMD and 75% AMD were placed on the left side of the vertical line, representing PC1, whereas samples irrigated with control and 50% AMD were placed on the right. The separation observed in PLS-DA can be further explained in terms of the identified compounds using loading plots. All seventy-two samples were analyzed and no clear separation was achieved for both the 2D and 3D (Figure 5B,C) plots. The samples did not separate, indicating that they were related in composition.

Allantoin metabolites proved to have the highest peak area (4,658,393) in Fianna when irrigated with 1:3 treatment. Creatine, niacinamide, adenosine, pyruvic acid, and citric acid were released in highest concentrations of 17,997,745, 194,152, 2,117,310, 9,305,996, and 4057 for Fianna cultivar; and recorded 3095, 199,0194, 690,607, 4,927,739, and 4,314,521 for Lady Rosetta, respectively, by plants irrigated with the 1:0 treatments relative to the concentrations of the same metabolites produced by plants watered with the same treatments. The production of the highest concentrations of amino acids by the plants irrigated with the 1:3 solutions might have been caused by the stress response induced in plants by the salts precipitated in the 1:1 irrigation treatment. This treatment contained salts, such as sulfate and chloride, with the highest concentrations of 2729.91 and 25.24 mg/kg, respectively compared to the other solutions used for the irrigation of crops. The plants in the plot irrigated with the 3:1 treatment had the second-highest concentrations of amino acids compared to amino acid concentrations achieved by plants irrigated with the 1:3 treatments.

Figure S2 depicts the correlation between primary metabolites and different FA:AMD treatments. Malic acid was recorded as three times higher in untreated AMD compared to the control. The above finding is supported by research by Shulaev et al. [81], who treated *Silene cucubalus* cell cultures with cadmium. Malic acid and acetate were shown to have increased levels in cadmium-treated cells, while glutamate and branched-chain amino acids decreased. Figure S2 shows a data matrix where coloring gives an overview of the numeric differences. In MetaboAnalyst, hierarchical clustering can be optionally applied to dimensions and/or observations. Ordering of the clustering tree can be configured and annotation tracks can be placed at the top of the matrix to interpret them in conjunction with the clustering tree. A good color scheme is an essential factor for the correct interpretation of a heatmap. Diverging palettes fix the color at both the lower and higher ends of the data, and the middle. They are better suited for data that range in both negative as well as positive directions. Sequential palettes fix the lowest and the highest values; they are more appropriate for non-negative data. Figure 2A results show different clusters for potato cultivars irrigated with different levels of FA:AMD treatment. Figure 5. further stipulates different clusters for potato cultivars irrigated with different levels of FA:AMD treatment. Nicotinic acid content was remarkably higher in samples 1, 21, 23, and 14; however, the same compounds were lower in potato samples 22, 7, 13, and 9, respectively. 2-Ketoglutaric acid was recorded as highest in samples 1, 14, and 12; whereas it was low in 6, 9, 21, and 24. Heavy metals are linked to amino acids like glycine, glutamine, serine, methionine, lysine, arginine, and proline, whereas ATP production is linked to metabolites like adenosine, adenine, and guanine [82]. Furthermore, during Cr stress, the expression of glutamine synthesis (GS) is increased; this enzyme is important for the maintenance of tissue antioxidant capabilities and the regulation of redox-sensitive signaling [83].

## 4. Discussion

Figure 1A (Supplementary Data) depicts ^1^H NMR spectra (superimposed) of *Solanum tuberosum* L. irrigated with treatment one (control) and four (untreated AMD). The results show that potatoes irrigated with treatment four (untreated AMD) had significantly higher concentrations of metabolites compared to the control. The metabolites at 8.5 ppm are among the peaks that prove that the peaks irrigated with untreated showed higher concentration of metabolites; the concentration of the metabolite roughly tripled the concentration when untreated AMD was applied. The same trend is observed in the sugar region between 3.2 to 4.2 ppm in the ^1^H spectra. Additionally, the doubled spectra at 1.5 and 3.65 ppm show that the concentration of metabolites irrigated with untreated AMD increased significantly when compared to the control. The above findings are supported by [84]; in their study, potatoes were grown under stressed abiotic conditions (plants were cultivated in Cd, As, and Cd^+^ environments). Sucrose, fructose, and glucose contents in leaves, roots, and stolons increased considerably in response to Cd and/or As stress when compared to the control. Plant hormones, such as auxin, brassinolide, abscisic acid, salicylic acid, and other hormones, were reported to be primary messengers in plant adaptation to stressful situations in several studies [44,85,86].

Other work [79,87,88] confirmed the findings of the current study. In that work, purple sweet potato was exposed to uranium (U) and cadmium (Cd) toxicity, and the physiological response and metabolomic networks were analyzed. Metabolites in the primary metabolic networks (e.g., carbohydrates, amino acids, lipids) were generally considerably elevated. Maltotriose, myo-inositol, melibiose, neohesperidose, and lactulose expression in the roots of the U and U^+^ Cd exposure groups were considerably higher than in the control. Furthermore, after U exposure, the glucose and glucono-1,5-lactone contents in the roots increased considerably. The plants’ heavy metal stress response methods were reflected in the metabolite regulation network [71,72].

More studies [76,89,90,91] also emphasized that metabolomics is now a crucial tool in the selection of plants that are resistant to changing climatic circumstances. Drought, salinity, soil floods, and severe temperatures are only a few of the abiotic stress conditions that produce major alterations in the plant metabolome. In addition, to maintain survival under severe environmental conditions, plants develop numerous adaptive strategies to endure abiotic stresses, including modifications in metabolism in different conditions. Nonetheless, the concentrations of some of the carbohydrates, including sucrose, raffinose, glucose, fructose, and maltose, increased, whereas the myoinositol level decreased in water-stressed barley roots. It was confirmed by [92] that metal ions (lanthanum, europium, silver, and cadmium) influenced secondary metabolite production. The effective accumulation of metals (Cr, Fe, Zn, and Mn) resulted in a 35 percent increase in oil content in *Brassica juncea*. Cu^2+^ and Cd^2+^ have been proven to increase the yields of secondary metabolites, such as shikonin, as well as digitalin synthesis. In *Beta vulgaris*, Cu^2+^ boosted the formation of betalains. Co^2+^ and Cu^2+^ have a stimulatory influence on secondary metabolite synthesis.

The LC-MS/MS Triple Quadrupole detected different groups of primary metabolites and statistical analysis of their integrated peak revealed significant differences among FA:AMD treatments. Different AMD treatments have significantly affected both Fianna and Lady Rosetta cultivars. Untreated AMD showed, significantly, the highest argininosuccinic acid peak area of 14,285,023 and 6,914,424 for Lady Rosetta and Fianna, respectively. It is confirmed in Figure S2 that potatoes watered with the 1:3 treatments displayed the highest concentrations of amino acids that were significantly different from amino acids concentrations produced by plants irrigated with either tap water or untreated AMD or the FA-treated AMD treatments. Exposure of plants to salt stress can accumulate organic osmolytes, including argininosuccinic acid, proline, valine, isoleucine, aspartic acid, betaine, glucose, fructose, sucrose, fructans, mannitol, pinitol, and inositol, in the cytoplasm of their cells [93].

Other researchers [92,93] outlined that chromium exposure causes metabolic disturbances, such as pigment change, elevated metabolite production (ascorbic acid, glutathione), and the formation of new metabolites as a reinforcement to the plant’s detoxification mechanism, whereas nickel toxicity can cause growth suppression and Fe levels to drop. Even if an amount of these trace elements can be filtered or rejected through plant root tissues, higher levels of heavy metals in soil are affiliated with virulent effects [94]. As has been observed in this study, where potato crops were irrigated with untreated AMD, heavy metals stimulated the biosynthesis of primary metabolites, such as adenine, aspartic acid, alanine, and 2-ketoglutaric acid metabolite, and the activity of antioxidant enzymes (catalase and peroxidase).

Moreover, the levels of the same parameters of potato samples irrigated with FA:AMD ratios had more concentration of metabolites when compared to samples irrigated with untreated AMD when compared to the control. Given that potatoes are considered staple foods, contamination of these food tubers by heavy metals, not only reduces their nutritional value, but also causes a considerable risk. Therefore, legal limits of heavy metals in food need to be revised and introduced by competent legislative authorities worldwide. Figure 2A and Figure 3A illustrate the correlation between the metabolites found in potato samples irrigated with treated and untreated AMD. There is a high correlation when creatine is compared to creatine, hence the darker red color; on the other hand, creatine shows no correlation when compared to 5-glutamylcysteine, glutamic acid, proline, thymidine, tyrosine, asparagine, aspartic acid, argininosuccinic acid, pantothenic acid, norepinephrine, and citric acid. There is a slight correlation between creatine and serine, glycine, threonine, malic acid, and adenine.

## 5. Conclusions

The NMR spectra appeared as a complex collective of resonances, representing the presence of a multitude of metabolites. In addition, given the occurrence of resonances throughout the whole spectral width (0–10 ppm), variable chemical features ranging from aliphatic to aromatic groups were detected in the samples for all treatments. Above all, strong signals in the sugar region in all treatments were observed, indicating the presence of free sugars. From a visual comparison of the spectra, there is an obvious similarity between all spectra, suggesting similar metabolic profiles between all AMD-treated samples, thus, allowing for the identification of primary metabolites, such as sugars, amino acids, and organic acids. Based on these NMR profiles, the relative variations between samples treated with AMD were visualized by plotting the scores of principal component analyses. Based on a multivariate statistical analysis of ^1^H NMR-based metabolomics data, extracts of two potato cultivars irrigated with varying FA:AMD ratios were compared and characterized in this study. A vast number of primary metabolites (sugars) and a small number of secondary metabolites (phenolics) were discovered. The metabolite profiles of *Solanum tuberosum* L. irrigated with various amounts of FA:AMD showed substantial variations, indicating that multiple metabolites can be utilized as origin identifiers. The metabolites showed the greatest and most significant variations in the samples irrigated with untreated AMD, particularly the sugar area impacted by glycosylation. The quantities of phenolic metabolites in treatment three and treatment four were relatively high. As a result, while there were many similarities, there were other compounds that were distinct, and were irrigated with various FA:AMD ratios.

To better understand the differences and similarities, more sensitive techniques, such as LC-MS, should be utilized to discover relatively low-abundance secondary metabolites that could be responsible for observed differences. We showed in this study that ^1^H NMR-based profiling is a quick and efficient way to fingerprint metabolic variations between *Solanum tuberosum* L. cultivars irrigated with different AMD treatments. This analytical method may be used to distinguish between *Solanum tuberosum* L origins and to identify the primary and secondary metabolites that are responsible for differentiation. The next stage in this research is to use LC-MS to analyze the same samples used in this research to find more secondary metabolites.

Metabolic profiling and metabolomic techniques are reliable and well-studied investigative tools. In this study, methanol extracts of *Solanum tuberosum* L. irrigated with different levels of AMD were compared and characterized based on a multivariate statistical analysis of LC-MS data. Because of its great sensitivity, LC-MS detected mostly secondary metabolites. Many of the metabolites could not be accurately annotated, thus further analysis is necessary. For a more detailed annotation of all metabolites, MS/MS was carried out in which fragmentation of a parent ion mass was performed. Overall, LC-MS was sensitive enough to detect the less-abundant secondary metabolites from *Solanum tuberosum* L. tubers, which could not be easily detected by NMR. This study serves to provide first-hand information and can thus be used as a benchmark for further studies.

## Data Availability

Not applicable.

## Supplementary Materials

The following are available online. Table S1. The *Solanum tuberosum* L. metabolites of samples irrigated with treated and untreated AMD. The retention times (min), compounds name, and accurate masses (*m*/*z*). Figure S1. 1H NMR spectra (superimposed) of *Solanum tuberosum* L. irrigated with treatment one and four. Figure S2. The heatmap for FA:AMD treatments on *Solanum tuberosum* L. samples primary metabolites identified from LC-MS/MS (Liquid Chromatography with Tandem Mass Spectrometry) triple quadrupole using unsupervised hierarchical clustering. The row represents the primary metabolites and the column represents samples irrigated with FA:AMD treatments. Figure S3. A correlation heatmap produced by MetaboAnalyst for *Solanum tuberosum* L. samples irrigated with treated and untreated AMD during the growing season. Red squares in the heatmap indicate increased excretion, while blue squares indicate decreased excretion.

## Sample Availability

Not available.
