# Supplementary material for: 1H-NMR and LC-MS Based Metabolomics Analysis of Potato (Solanum tuberosum L.) Cultivars Irrigated with Fly Ash Treated Acid Mine Drainage
Source: Molecules. 2022 Feb 10;27(4):1187. doi: 10.3390/molecules27041187 (PMC8877823; doi:10.3390/molecules27041187)
Supplement: Supplementary file 1 [file molecules-27-01187-s001.zip › molecules-1540912-supplementary.pdf]

Table S1. The *Solanum tuberosum* L. metabolites of samples irrigated with treated and untreated AMD. The retention times (min), compounds name, and accurate masses (m/z).

Peaks	Compound Name	Molecular formula	r_T (min)	Accurate mass (m/z)	Literature
**1**	Calystegine A3	$C_7H_{13}NO_3$	0.79	160.0969	Petersson *et al.* 2013
**2**	Quinic acid	$C_7H_{12}O_6$	0.95	193.0342	Villegas and Kojima, 1985
**3**	Kukoamine A	$C_{28}H_{42}N_4O_6$	4.21	531.3184	Funayama *et al.* 1980
**4**	Chlorogenic acid	$C_{16}H_{18}O_9$	4.89	355.1030	Villegas and Kojima, 1985
**5**	Caffeic acid	$C_9H_8O_4$	5.36	181.0497	Mattila and Hellström, 2007
**6**	Rutin	$C_{27}H_{30}O_{16}$	6.53	611.1616	Oliveira *et al.* 2013
**7**	Chaconine	$C_{45}H_{73}NO_{14}$	7.02	852.5094	Duggan *et al.* 2020
**8**	Solanidine	$C_{27}H_{43}NO$	7.06	398.3419	Wang et al. 2020
**9**	Solanine	$C_{45}H_{73}NO_{15}$	7.07	868.5056	Duggan *et al.* 2020
**10**	Azelaic acid	$C_9H_{16}O_4$	7.29	189.1129	Martins *et al.* 2016
**11**	Limonene	$C_{10}H_{16}$	10.39	137.1325	Schieberle *et al.* 1987
**12**	Oleamide	$C_{18}H_{35}NO$	11.44	282.2788	Cravatt *et al.* 1996
**13**	Linolenic acid	$C_{18}H_{30}O_2$	11.61	281.2476	Alexandri *et al.* 2012

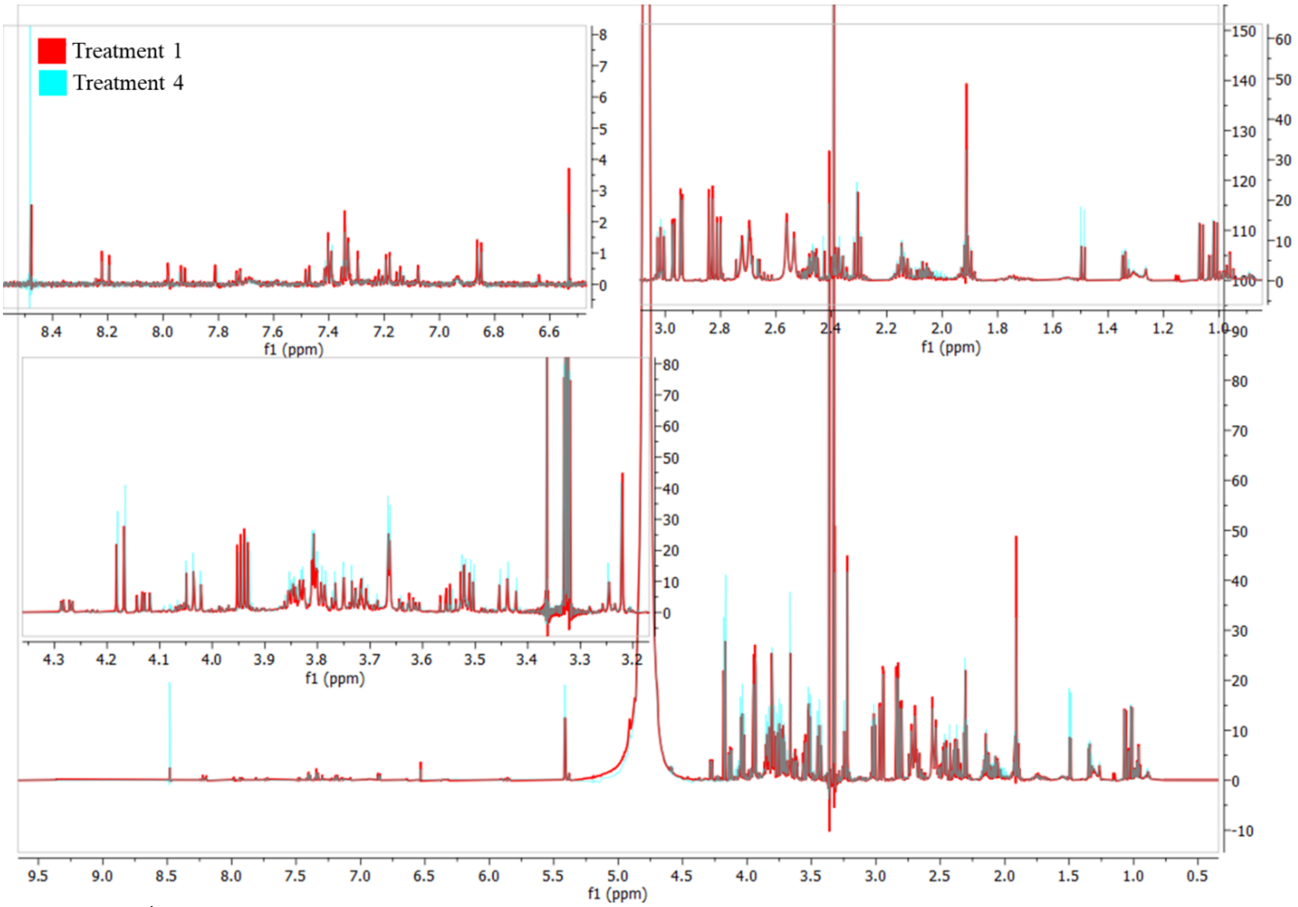

Figure S1. ^{1}H NMR spectra (superimposed) of *Solanum tuberosum* L. irrigated with treatment 1 and 4.

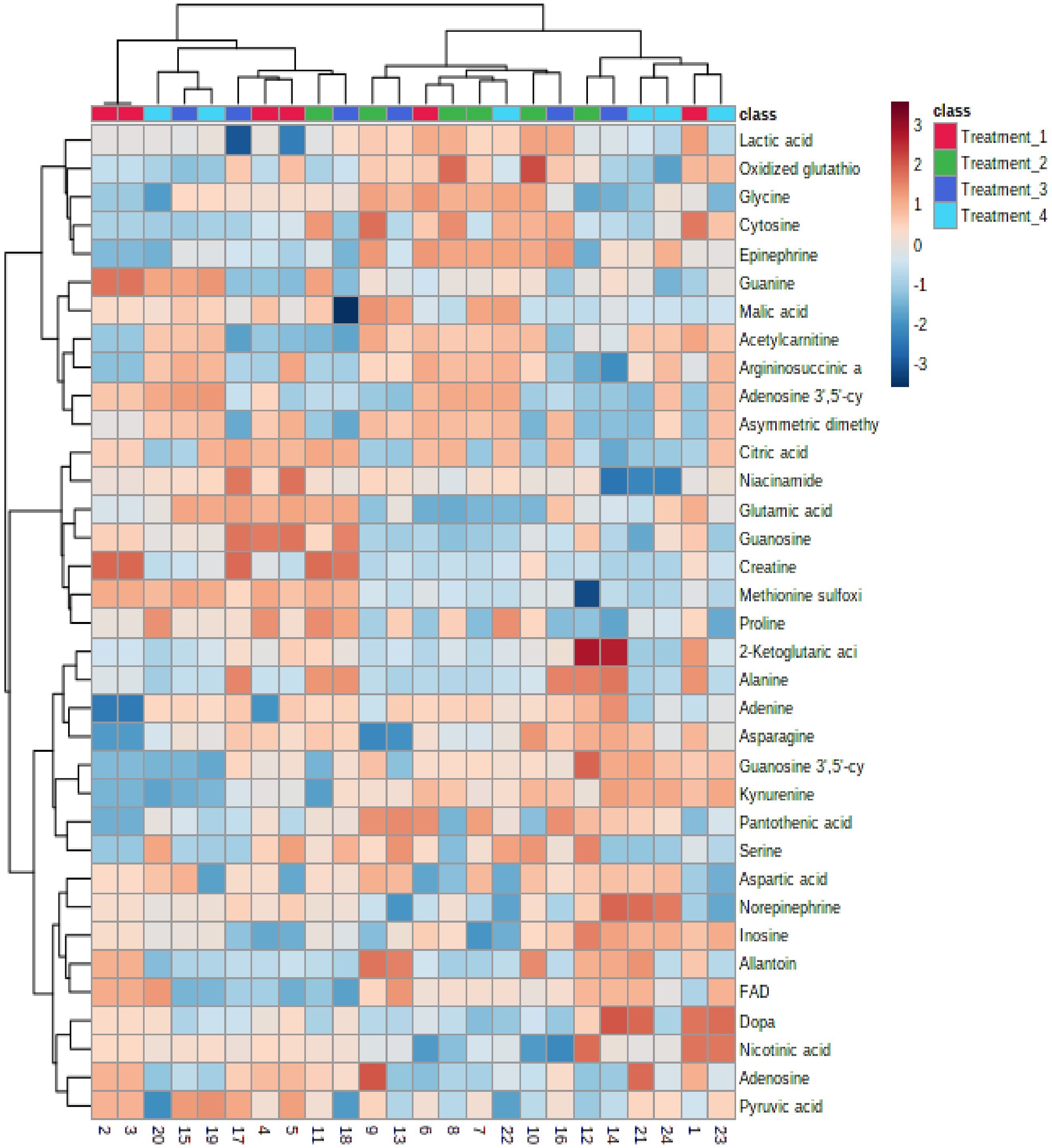

Figure S2. The heatmap for FA: AMD treatments on *Solanum tuberosum* L. samples primary metabolites identified from LC-MS/MS (Liquid Chromatography with Tandem Mass Spectrometry) triple quadrupole using unsupervised hierarchical clustering. The row represents the primary metabolites and the column represents samples irrigated with FA: AMD treatments.

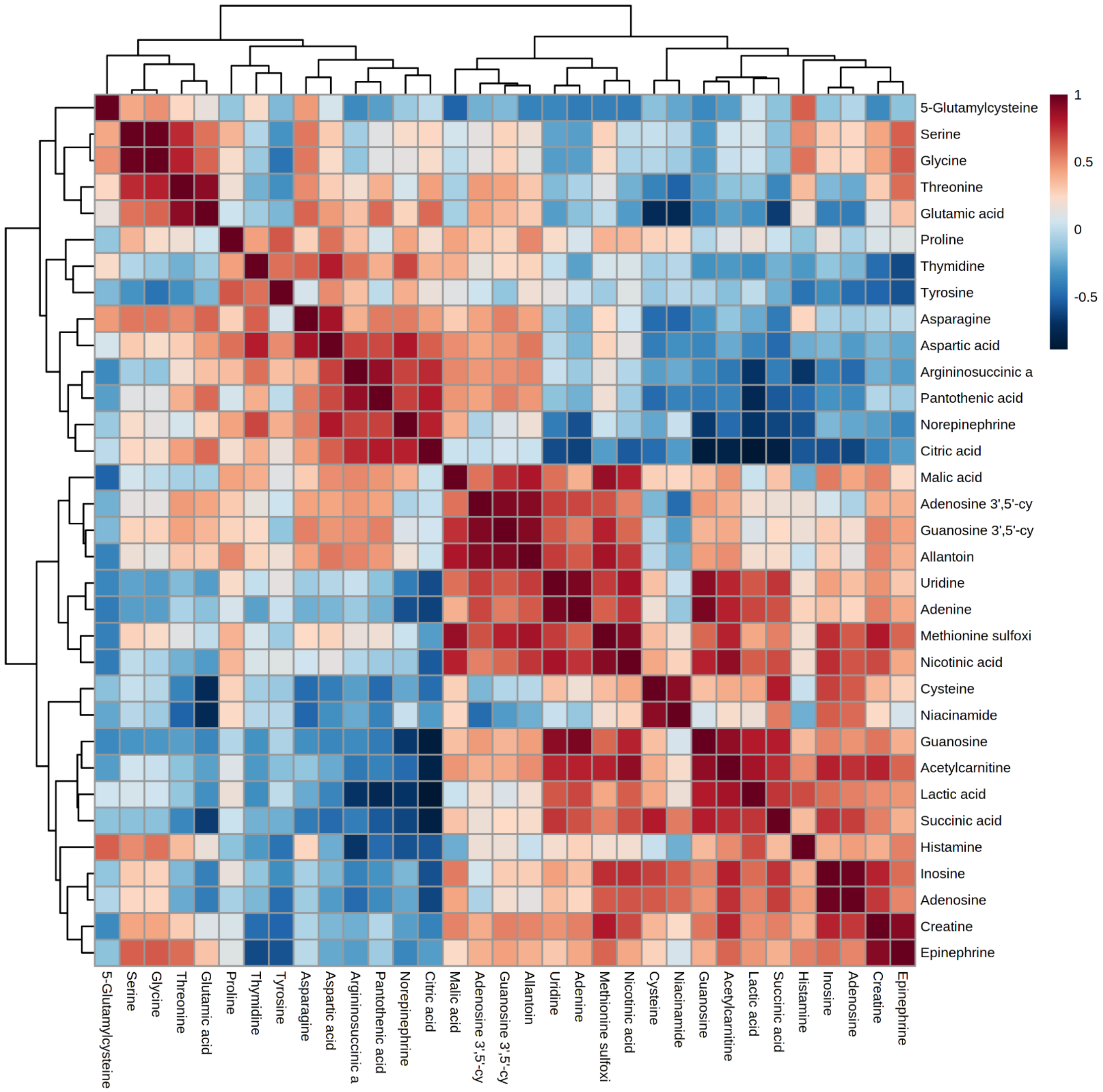

Figure S3. A correlation heatmap produced by MetaboAnalyst for *Solanum tuberosum* L. samples irrigated with treated and untreated AMD during the growing season. Red squares in the heatmap indicate increased excretion, while blue squares indicate decreased excretion.
